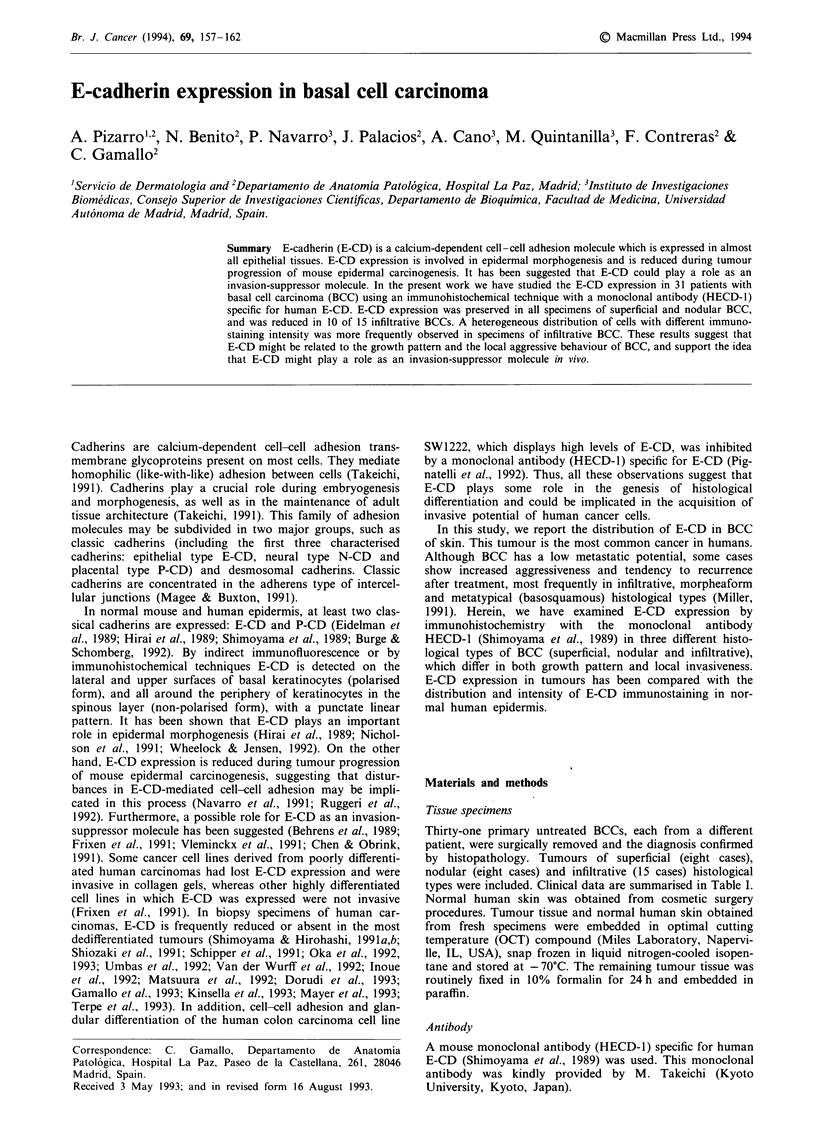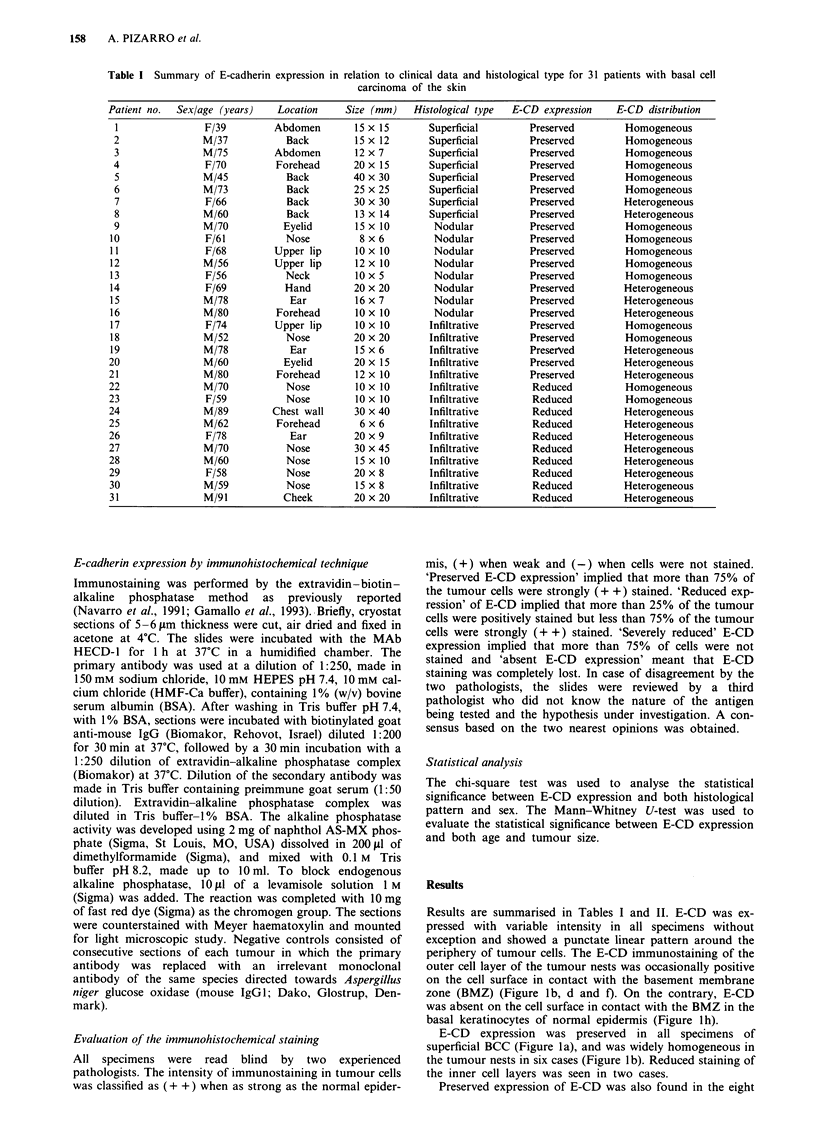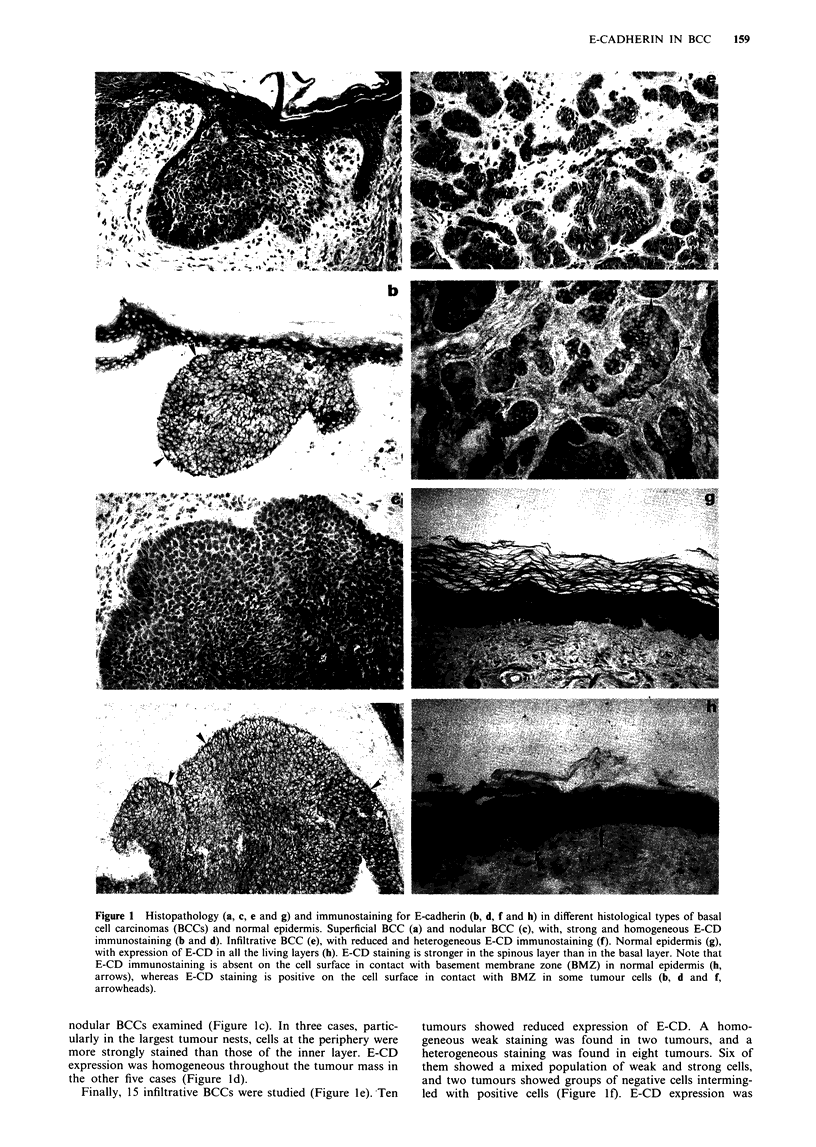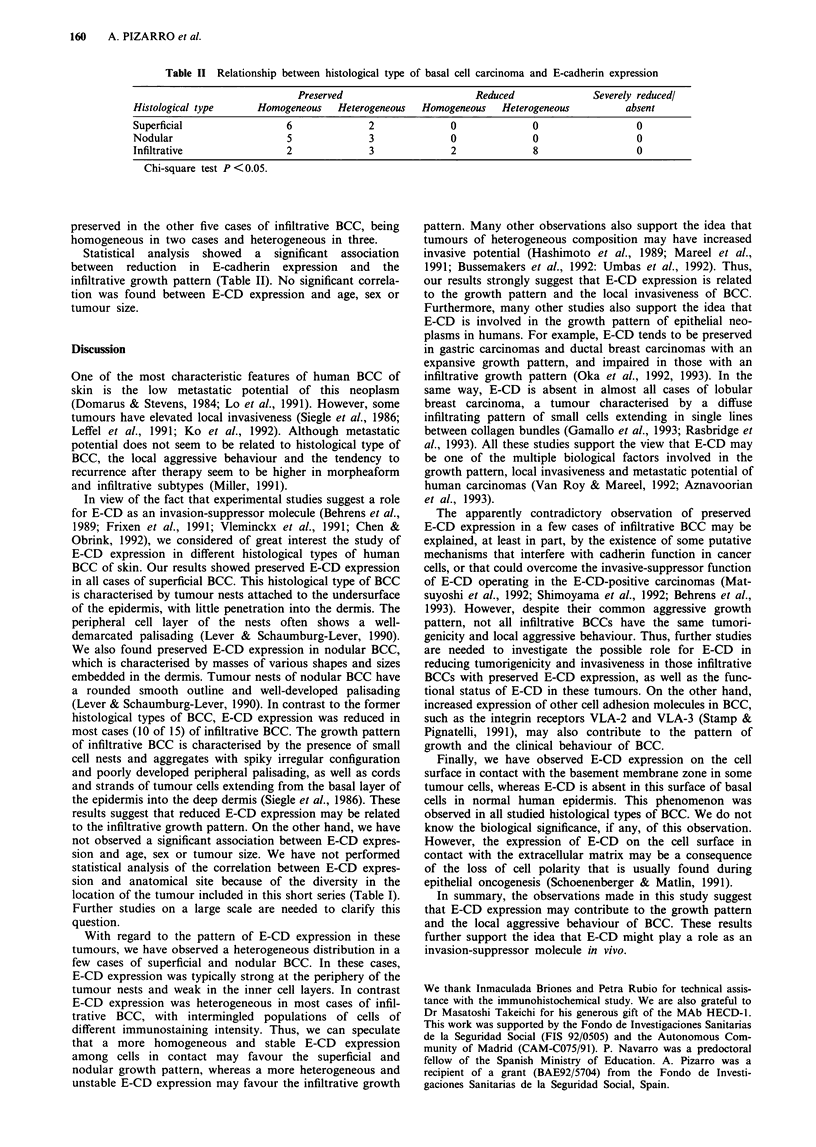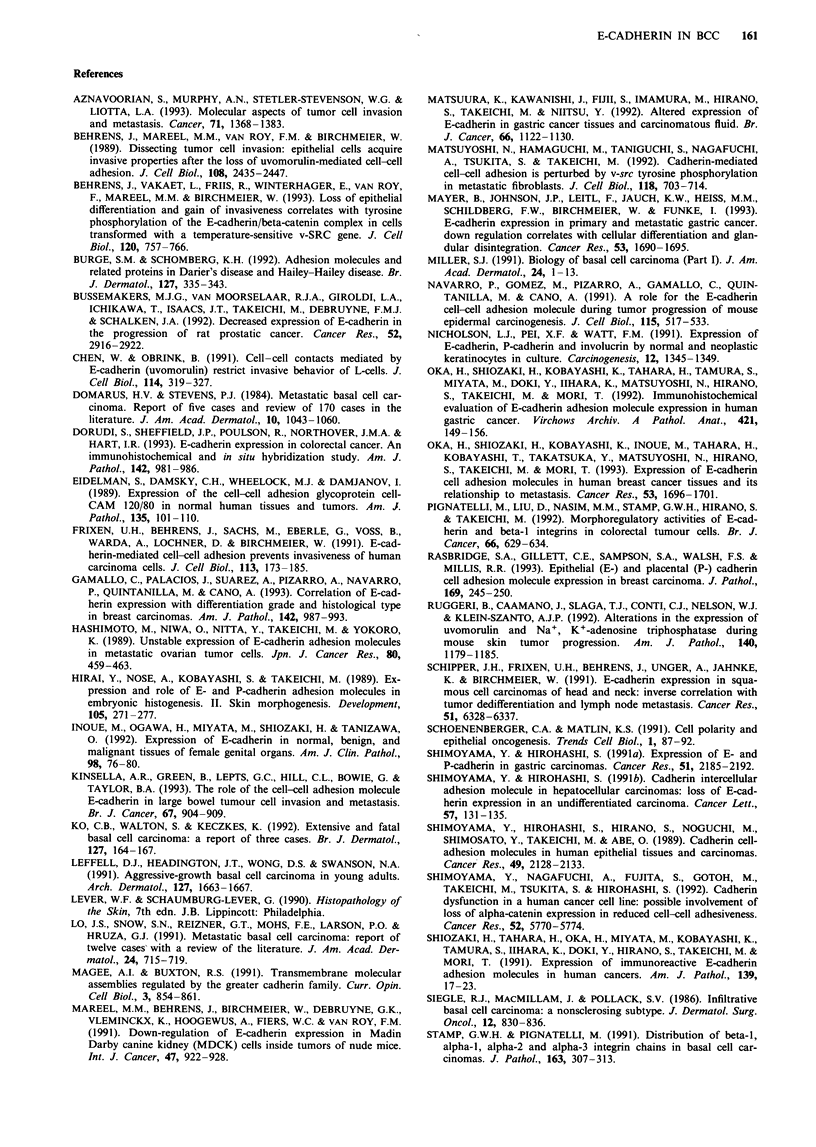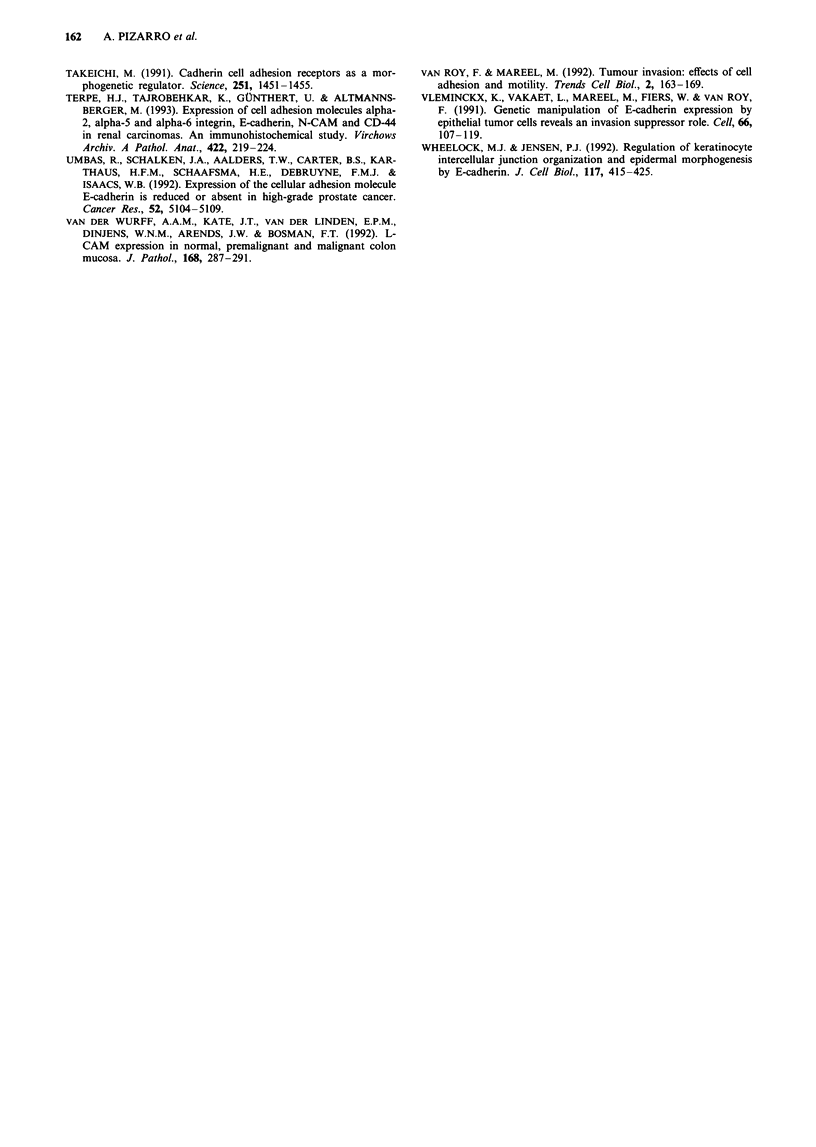# E-cadherin expression in basal cell carcinoma.

**DOI:** 10.1038/bjc.1994.26

**Published:** 1994-01

**Authors:** A. Pizarro, N. Benito, P. Navarro, J. Palacios, A. Cano, M. Quintanilla, F. Contreras, C. Gamallo

**Affiliations:** Servicio de Dermatologia, Hospital La Paz, Madrid, Spain.

## Abstract

**Images:**


Br. J. Cancer (1994), 69, 157-162 © Macmillan Press Ltd., 1994

### E-cadherin expression in basal cell carcinoma

A. Pizarro[1,2], N. Benito[2], P. Navarro[3], J. Palacios[2], A. Cano[3], M. Quintanilla[3], F. Contreras[2] & C. Gamallo[2]

[1]*Servicio de Dermatologia and* [2]*Departamento de Anatomía Patológica, Hospital La Paz, Madrid;* [3]*Instituto de Investigaciones Biomédicas, Consejo Superior de Investigaciones Científicas, Departamento de Bioquímica, Facultad de Medicina, Universidad Autónoma de Madrid, Madrid, Spain.*

**Summary** E-cadherin (E-CD) is a calcium-dependent cell-cell adhesion molecule which is expressed in almost all epithelial tissues. E-CD expression is involved in epidermal morphogenesis and is reduced during tumour progression of mouse epidermal carcinogenesis. It has been suggested that E-CD could play a role as an invasion-suppressor molecule. In the present work we have studied the E-CD expression in 31 patients with basal cell carcinoma (BCC) using an immunohistochemical technique with a monoclonal antibody (HECD-1) specific for human E-CD. E-CD expression was preserved in all specimens of superficial and nodular BCC, and was reduced in 10 of 15 infiltrative BCCs. A heterogeneous distribution of cells with different immunostaining intensity was more frequently observed in specimens of infiltrative BCC. These results suggest that E-CD might be related to the growth pattern and the local aggressive behaviour of BCC, and support the idea that E-CD might play a role as an invasion-suppressor molecule *in vivo*.

Cadherins are calcium-dependent cell-cell adhesion transmembrane glycoproteins present on most cells. They mediate homophilic (like-with-like) adhesion between cells (Takeichi, 1991). Cadherins play a crucial role during embryogenesis and morphogenesis, as well as in the maintenance of adult tissue architecture (Takeichi, 1991). This family of adhesion molecules may be subdivided in two major groups, such as classic cadherins (including the first three characterised cadherins: epithelial type E-CD, neural type N-CD and placental type P-CD) and desmosomal cadherins. Classic cadherins are concentrated in the adherens type of intercellular junctions (Magee & Buxton, 1991).

In normal mouse and human epidermis, at least two classical cadherins are expressed: E-CD and P-CD (Eidelman *et al.*, 1989; Hirai *et al.*, 1989; Shimoyama *et al.*, 1989; Burge & Schomberg, 1992). By indirect immunofluorescence or by immunohistochemical techniques E-CD is detected on the lateral and upper surfaces of basal keratinocytes (polarised form), and all around the periphery of keratinocytes in the spinous layer (non-polarised form), with a punctate linear pattern. It has been shown that E-CD plays an important role in epidermal morphogenesis (Hirai *et al.*, 1989; Nicholson *et al.*, 1991; Wheelock & Jensen, 1992). On the other hand, E-CD expression is reduced during tumour progression of mouse epidermal carcinogenesis, suggesting that disturbances in E-CD-mediated cell-cell adhesion may be implicated in this process (Navarro *et al.*, 1991; Ruggeri *et al.*, 1992). Furthermore, a possible role for E-CD as an invasion-suppressor molecule has been suggested (Behrens *et al.*, 1989; Frixen *et al.*, 1991; Vleminckx *et al.*, 1991; Chen & Obrink, 1991). Some cancer cell lines derived from poorly differentiated human carcinomas had lost E-CD expression and were invasive in collagen gels, whereas other highly differentiated cell lines in which E-CD was expressed were not invasive (Frixen *et al.*, 1991). In biopsy specimens of human carcinomas, E-CD is frequently reduced or absent in the most dedifferentiated tumours (Shimoyama & Hirohashi, 1991*a*,*b*; Shiozaki *et al.*, 1991; Schipper *et al.*, 1991; Oka *et al.*, 1992, 1993; Umbas *et al.*, 1992; Van der Wurff *et al.*, 1992; Inoue *et al.*, 1992; Matsuura *et al.*, 1992; Dorudi *et al.*, 1993; Gamallo *et al.*, 1993; Kinsella *et al.*, 1993; Mayer *et al.*, 1993; Terpe *et al.*, 1993). In addition, cell-cell adhesion and glandular differentiation of the human colon carcinoma cell line SW1222, which displays high levels of E-CD, was inhibited by a monoclonal antibody (HECD-1) specific for E-CD (Pignatelli *et al.*, 1992). Thus, all these observations suggest that E-CD plays some role in the genesis of histological differentiation and could be implicated in the acquisition of invasive potential of human cancer cells.

In this study, we report the distribution of E-CD in BCC of skin. This tumour is the most common cancer in humans. Although BCC has a low metastatic potential, some cases show increased aggressiveness and tendency to recurrence after treatment, most frequently in infiltrative, morpheaform and metatypical (basosquamous) histological types (Miller, 1991). Herein, we have examined E-CD expression by immunohistochemistry with the monoclonal antibody HECD-1 (Shimoyama *et al.*, 1989) in three different histological types of BCC (superficial, nodular and infiltrative), which differ in both growth pattern and local invasiveness. E-CD expression in tumours has been compared with the distribution and intensity of E-CD immunostaining in normal human epidermis.

### Materials and methods

### Tissue specimens

Thirty-one primary untreated BCCs, each from a different patient, were surgically removed and the diagnosis confirmed by histopathology. Tumours of superficial (eight cases), nodular (eight cases) and infiltrative (15 cases) histological types were included. Clinical data are summarised in Table I. Normal human skin was obtained from cosmetic surgery procedures. Tumour tissue and normal human skin obtained from fresh specimens were embedded in optimal cutting temperature (OCT) compound (Miles Laboratory, Naperville, IL, USA), snap frozen in liquid nitrogen-cooled isopentane and stored at $-70°C$. The remaining tumour tissue was routinely fixed in 10% formalin for 24 h and embedded in paraffin.

### Antibody

A mouse monoclonal antibody (HECD-1) specific for human E-CD (Shimoyama *et al.*, 1989) was used. This monoclonal antibody was kindly provided by M. Takeichi (Kyoto University, Kyoto, Japan).

Correspondence: C. Gamallo, Departamento de Anatomía Patológica, Hospital La Paz, Paseo de la Castellana, 261, 28046 Madrid, Spain.
Received 3 May 1993; and in revised form 16 August 1993.

**Table I** Summary of E-cadherin expression in relation to clinical data and histological type for 31 patients with basal cell carcinoma of the skin

| *Patient no.* | *Sex/age (years)* | *Location* | *Size (mm)* | *Histological type* | *E-CD expression* | *E-CD distribution* |
|---|---|---|---|---|---|---|
| 1 | F/39 | Abdomen | 15 × 15 | Superficial | Preserved | Homogeneous |
| 2 | M/37 | Back | 15 × 12 | Superficial | Preserved | Homogeneous |
| 3 | M/75 | Abdomen | 12 × 7 | Superficial | Preserved | Homogeneous |
| 4 | F/70 | Forehead | 20 × 15 | Superficial | Preserved | Homogeneous |
| 5 | M/45 | Back | 40 × 30 | Superficial | Preserved | Homogeneous |
| 6 | M/73 | Back | 25 × 25 | Superficial | Preserved | Homogeneous |
| 7 | F/66 | Back | 30 × 30 | Superficial | Preserved | Heterogeneous |
| 8 | M/60 | Back | 13 × 14 | Superficial | Preserved | Heterogeneous |
| 9 | M/70 | Eyelid | 15 × 10 | Nodular | Preserved | Homogeneous |
| 10 | F/61 | Nose | 8 × 6 | Nodular | Preserved | Homogeneous |
| 11 | F/68 | Upper lip | 10 × 10 | Nodular | Preserved | Homogeneous |
| 12 | M/56 | Upper lip | 12 × 10 | Nodular | Preserved | Homogeneous |
| 13 | F/56 | Neck | 10 × 5 | Nodular | Preserved | Homogeneous |
| 14 | F/69 | Hand | 20 × 20 | Nodular | Preserved | Heterogeneous |
| 15 | M/78 | Ear | 16 × 7 | Nodular | Preserved | Heterogeneous |
| 16 | M/80 | Forehead | 10 × 10 | Nodular | Preserved | Heterogeneous |
| 17 | F/74 | Upper lip | 10 × 10 | Infiltrative | Preserved | Homogeneous |
| 18 | M/52 | Nose | 20 × 20 | Infiltrative | Preserved | Homogeneous |
| 19 | M/78 | Ear | 15 × 6 | Infiltrative | Preserved | Heterogeneous |
| 20 | M/60 | Eyelid | 20 × 15 | Infiltrative | Preserved | Heterogeneous |
| 21 | M/80 | Forehead | 12 × 10 | Infiltrative | Preserved | Heterogeneous |
| 22 | M/70 | Nose | 10 × 10 | Infiltrative | Reduced | Homogeneous |
| 23 | F/59 | Nose | 10 × 10 | Infiltrative | Reduced | Homogeneous |
| 24 | M/89 | Chest wall | 30 × 40 | Infiltrative | Reduced | Heterogeneous |
| 25 | M/62 | Forehead | 6 × 6 | Infiltrative | Reduced | Heterogeneous |
| 26 | F/78 | Ear | 20 × 9 | Infiltrative | Reduced | Heterogeneous |
| 27 | M/70 | Nose | 30 × 45 | Infiltrative | Reduced | Heterogeneous |
| 28 | M/60 | Nose | 15 × 10 | Infiltrative | Reduced | Heterogeneous |
| 29 | F/58 | Nose | 20 × 8 | Infiltrative | Reduced | Heterogeneous |
| 30 | M/59 | Nose | 15 × 8 | Infiltrative | Reduced | Heterogeneous |
| 31 | M/91 | Cheek | 20 × 20 | Infiltrative | Reduced | Heterogeneous |

*E-cadherin expression by immunohistochemical technique*

Immunostaining was performed by the extravidin–biotin–alkaline phosphatase method as previously reported (Navarro *et al.*, 1991; Gamallo *et al.*, 1993). Briefly, cryostat sections of 5–6 μm thickness were cut, air dried and fixed in acetone at 4°C. The slides were incubated with the MAb HECD-1 for 1 h at 37°C in a humidified chamber. The primary antibody was used at a dilution of 1:250, made in 150 mM sodium chloride, 10 mM HEPES pH 7.4, 10 mM calcium chloride (HMF-Ca buffer), containing 1% (w/v) bovine serum albumin (BSA). After washing in Tris buffer pH 7.4, with 1% BSA, sections were incubated with biotinylated goat anti-mouse IgG (Biomakor, Rehovot, Israel) diluted 1:200 for 30 min at 37°C, followed by a 30 min incubation with a 1:250 dilution of extravidin–alkaline phosphatase complex (Biomakor) at 37°C. Dilution of the secondary antibody was made in Tris buffer containing preimmune goat serum (1:50 dilution). Extravidin–alkaline phosphatase complex was diluted in Tris buffer–1% BSA. The alkaline phosphatase activity was developed using 2 mg of naphthol AS-MX phosphate (Sigma, St Louis, MO, USA) dissolved in 200 μl of dimethylformamide (Sigma), and mixed with 0.1 M Tris buffer pH 8.2, made up to 10 ml. To block endogenous alkaline phosphatase, 10 μl of a levamisole solution 1 M (Sigma) was added. The reaction was completed with 10 mg of fast red dye (Sigma) as the chromogen group. The sections were counterstained with Meyer haematoxylin and mounted for light microscopic study. Negative controls consisted of consecutive sections of each tumour in which the primary antibody was replaced with an irrelevant monoclonal antibody of the same species directed towards *Aspergillus niger* glucose oxidase (mouse IgG1; Dako, Glostrup, Denmark).

*Evaluation of the immunohistochemical staining*

All specimens were read blind by two experienced pathologists. The intensity of immunostaining in tumour cells was classified as (+ +) when as strong as the normal epidermis, (+) when weak and (−) when cells were not stained. 'Preserved E-CD expression' implied that more than 75% of the tumour cells were strongly (+ +) stained. 'Reduced expression' of E-CD implied that more than 25% of the tumour cells were positively stained but less than 75% of the tumour cells were strongly (+ +) stained. 'Severely reduced' E-CD expression implied that more than 75% of cells were not stained and 'absent E-CD expression' meant that E-CD staining was completely lost. In case of disagreement by the two pathologists, the slides were reviewed by a third pathologist who did not know the nature of the antigen being tested and the hypothesis under investigation. A consensus based on the two nearest opinions was obtained.

*Statistical analysis*

The chi-square test was used to analyse the statistical significance between E-CD expression and both histological pattern and sex. The Mann–Whitney *U*-test was used to evaluate the statistical significance between E-CD expression and both age and tumour size.

### Results

Results are summarised in Tables I and II. E-CD was expressed with variable intensity in all specimens without exception and showed a punctate linear pattern around the periphery of tumour cells. The E-CD immunostaining of the outer cell layer of the tumour nests was occasionally positive on the cell surface in contact with the basement membrane zone (BMZ) (Figure 1b, d and f). On the contrary, E-CD was absent on the cell surface in contact with the BMZ in the basal keratinocytes of normal epidermis (Figure 1h).

E-CD expression was preserved in all specimens of superficial BCC (Figure 1a), and was widely homogeneous in the tumour nests in six cases (Figure 1b). Reduced staining of the inner cell layers was seen in two cases.

Preserved expression of E-CD was also found in the eight

**Figure 1** Histopathology (**a**, **c**, **e** and **g**) and immunostaining for E-cadherin (**b**, **d**, **f** and **h**) in different histological types of basal cell carcinomas (BCCs) and normal epidermis. Superficial BCC (**a**) and nodular BCC (**c**), with, strong and homogeneous E-CD immunostaining (**b** and **d**). Infiltrative BCC (**e**), with reduced and heterogeneous E-CD immunostaining (**f**). Normal epidermis (**g**), with expression of E-CD in all the living layers (**h**). E-CD staining is stronger in the spinous layer than in the basal layer. Note that E-CD immunostaining is absent on the cell surface in contact with basement membrane zone (BMZ) in normal epidermis (**h**, arrows), whereas E-CD staining is positive on the cell surface in contact with BMZ in some tumour cells (**b**, **d** and **f**, arrowheads).

nodular BCCs examined (Figure 1c). In three cases, particularly in the largest tumour nests, cells at the periphery were more strongly stained than those of the inner layer. E-CD expression was homogeneous throughout the tumour mass in the other five cases (Figure 1d).

Finally, 15 infiltrative BCCs were studied (Figure 1e). Ten tumours showed reduced expression of E-CD. A homogeneous weak staining was found in two tumours, and a heterogeneous staining was found in eight tumours. Six of them showed a mixed population of weak and strong cells, and two tumours showed groups of negative cells intermingled with positive cells (Figure 1f). E-CD expression was

**Table II** Relationship between histological type of basal cell carcinoma and E-cadherin expression

| | *Preserved* | | *Reduced* | | *Severely reduced/* |
|---|---|---|---|---|---|
| *Histological type* | *Homogeneous* | *Heterogeneous* | *Homogeneous* | *Heterogeneous* | *absent* |
| Superficial | 6 | 2 | 0 | 0 | 0 |
| Nodular | 5 | 3 | 0 | 0 | 0 |
| Infiltrative | 2 | 3 | 2 | 8 | 0 |

Chi-square test $P < 0.05$.

preserved in the other five cases of infiltrative BCC, being homogeneous in two cases and heterogeneous in three.

Statistical analysis showed a significant association between reduction in E-cadherin expression and the infiltrative growth pattern (Table II). No significant correlation was found between E-CD expression and age, sex or tumour size.

### Discussion

One of the most characteristic features of human BCC of skin is the low metastatic potential of this neoplasm (Domarus & Stevens, 1984; Lo *et al.*, 1991). However, some tumours have elevated local invasiveness (Siegle *et al.*, 1986; Leffel *et al.*, 1991; Ko *et al.*, 1992). Although metastatic potential does not seem to be related to histological type of BCC, the local aggressive behaviour and the tendency to recurrence after therapy seem to be higher in morpheaform and infiltrative subtypes (Miller, 1991).

In view of the fact that experimental studies suggest a role for E-CD as an invasion-suppressor molecule (Behrens *et al.*, 1989; Frixen *et al.*, 1991; Vleminckx *et al.*, 1991; Chen & Obrink, 1992), we considered of great interest the study of E-CD expression in different histological types of human BCC of skin. Our results showed preserved E-CD expression in all cases of superficial BCC. This histological type of BCC is characterised by tumour nests attached to the undersurface of the epidermis, with little penetration into the dermis. The peripheral cell layer of the nests often shows a well-demarcated palisading (Lever & Schaumburg-Lever, 1990). We also found preserved E-CD expression in nodular BCC, which is characterised by masses of various shapes and sizes embedded in the dermis. Tumour nests of nodular BCC have a rounded smooth outline and well-developed palisading (Lever & Schaumburg-Lever, 1990). In contrast to the former histological types of BCC, E-CD expression was reduced in most cases (10 of 15) of infiltrative BCC. The growth pattern of infiltrative BCC is characterised by the presence of small cell nests and aggregates with spiky irregular configuration and poorly developed peripheral palisading, as well as cords and strands of tumour cells extending from the basal layer of the epidermis into the deep dermis (Siegle *et al.*, 1986). These results suggest that reduced E-CD expression may be related to the infiltrative growth pattern. On the other hand, we have not observed a significant association between E-CD expression and age, sex or tumour size. We have not performed statistical analysis of the correlation between E-CD expression and anatomical site because of the diversity in the location of the tumour included in this short series (Table I). Further studies on a large scale are needed to clarify this question.

With regard to the pattern of E-CD expression in these tumours, we have observed a heterogeneous distribution in a few cases of superficial and nodular BCC. In these cases, E-CD expression was typically strong at the periphery of the tumour nests and weak in the inner cell layers. In contrast E-CD expression was heterogeneous in most cases of infiltrative BCC, with intermingled populations of cells of different immunostaining intensity. Thus, we can speculate that a more homogeneous and stable E-CD expression among cells in contact may favour the superficial and nodular growth pattern, whereas a more heterogeneous and unstable E-CD expression may favour the infiltrative growth pattern. Many other observations also support the idea that tumours of heterogeneous composition may have increased invasive potential (Hashimoto *et al.*, 1989; Mareel *et al.*, 1991; Bussemakers *et al.*, 1992: Umbas *et al.*, 1992). Thus, our results strongly suggest that E-CD expression is related to the growth pattern and the local invasiveness of BCC. Furthermore, many other studies also support the idea that E-CD is involved in the growth pattern of epithelial neoplasms in humans. For example, E-CD tends to be preserved in gastric carcinomas and ductal breast carcinomas with an expansive growth pattern, and impaired in those with an infiltrative growth pattern (Oka *et al.*, 1992, 1993). In the same way, E-CD is absent in almost all cases of lobular breast carcinoma, a tumour characterised by a diffuse infiltrating pattern of small cells extending in single lines between collagen bundles (Gamallo *et al.*, 1993; Rasbridge *et al.*, 1993). All these studies support the view that E-CD may be one of the multiple biological factors involved in the growth pattern, local invasiveness and metastatic potential of human carcinomas (Van Roy & Mareel, 1992; Aznavoorian *et al.*, 1993).

The apparently contradictory observation of preserved E-CD expression in a few cases of infiltrative BCC may be explained, at least in part, by the existence of some putative mechanisms that interfere with cadherin function in cancer cells, or that could overcome the invasive-suppressor function of E-CD operating in the E-CD-positive carcinomas (Matsuyoshi *et al.*, 1992; Shimoyama *et al.*, 1992; Behrens *et al.*, 1993). However, despite their common aggressive growth pattern, not all infiltrative BCCs have the same tumorigenicity and local aggressive behaviour. Thus, further studies are needed to investigate the possible role for E-CD in reducing tumorigenicity and invasiveness in those infiltrative BCCs with preserved E-CD expression, as well as the functional status of E-CD in these tumours. On the other hand, increased expression of other cell adhesion molecules in BCC, such as the integrin receptors VLA-2 and VLA-3 (Stamp & Pignatelli, 1991), may also contribute to the pattern of growth and the clinical behaviour of BCC.

Finally, we have observed E-CD expression on the cell surface in contact with the basement membrane zone in some tumour cells, whereas E-CD is absent in this surface of basal cells in normal human epidermis. This phenomenon was observed in all studied histological types of BCC. We do not know the biological significance, if any, of this observation. However, the expression of E-CD on the cell surface in contact with the extracellular matrix may be a consequence of the loss of cell polarity that is usually found during epithelial oncogenesis (Schoenenberger & Matlin, 1991).

In summary, the observations made in this study suggest that E-CD expression may contribute to the growth pattern and the local aggressive behaviour of BCC. These results further support the idea that E-CD might play a role as an invasion-suppressor molecule *in vivo*.

We thank Inmaculada Briones and Petra Rubio for technical assistance with the immunohistochemical study. We are also grateful to Dr Masatoshi Takeichi for his generous gift of the MAb HECD-1. This work was supported by the Fondo de Investigaciones Sanitarias de la Seguridad Social (FIS 92/0505) and the Autonomous Community of Madrid (CAM-C075/91). P. Navarro was a predoctoral fellow of the Spanish Ministry of Education. A. Pizarro was a recipient of a grant (BAE92/5704) from the Fondo de Investigaciones Sanitarias de la Seguridad Social, Spain.

### References

AZNAVOORIAN, S., MURPHY, A.N., STETLER-STEVENSON, W.G. & LIOTTA, L.A. (1993). Molecular aspects of tumor cell invasion and metastasis. *Cancer*, **71**, 1368–1383.

BEHRENS, J., MAREEL, M.M., VAN ROY, F.M. & BIRCHMEIER, W. (1989). Dissecting tumor cell invasion: epithelial cells acquire invasive properties after the loss of uvomorulin-mediated cell–cell adhesion. *J. Cell Biol.*, **108**, 2435–2447.

BEHRENS, J., VAKAET, L., FRIIS, R., WINTERHAGER, E., VAN ROY, F., MAREEL, M.M. & BIRCHMEIER, W. (1993). Loss of epithelial differentiation and gain of invasiveness correlates with tyrosine phosphorylation of the E-cadherin/beta-catenin complex in cells transformed with a temperature-sensitive v-SRC gene. *J. Cell Biol.*, **120**, 757–766.

BURGE, S.M. & SCHOMBERG, K.H. (1992). Adhesion molecules and related proteins in Darier's disease and Hailey–Hailey disease. *Br. J. Dermatol.*, **127**, 335–343.

BUSSEMAKERS, M.J.G., VAN MOORSELAAR, R.J.A., GIROLDI, L.A., ICHIKAWA, T., ISAACS, J.T., TAKEICHI, M., DEBRUYNE, F.M.J. & SCHALKEN, J.A. (1992). Decreased expression of E-cadherin in the progression of rat prostatic cancer. *Cancer Res.*, **52**, 2916–2922.

CHEN, W. & OBRINK, B. (1991). Cell–cell contacts mediated by E-cadherin (uvomorulin) restrict invasive behavior of L-cells. *J. Cell Biol.*, **114**, 319–327.

DOMARUS, H.V. & STEVENS, P.J. (1984). Metastatic basal cell carcinoma. Report of five cases and review of 170 cases in the literature. *J. Am. Acad. Dermatol.*, **10**, 1043–1060.

DORUDI, S., SHEFFIELD, J.P., POULSON, R., NORTHOVER, J.M.A. & HART, I.R. (1993). E-cadherin expression in colorectal cancer. An immunohistochemical and *in situ* hybridization study. *Am. J. Pathol.*, **142**, 981–986.

EIDELMAN, S., DAMSKY, C.H., WHEELOCK, M.J. & DAMJANOV, I. (1989). Expression of the cell–cell adhesion glycoprotein cell-CAM 120/80 in normal human tissues and tumors. *Am. J. Pathol.*, **135**, 101–110.

FRIXEN, U.H., BEHRENS, J., SACHS, M., EBERLE, G., VOSS, B., WARDA, A., LOCHNER, D. & BIRCHMEIER, W. (1991). E-cadherin-mediated cell–cell adhesion prevents invasiveness of human carcinoma cells. *J. Cell Biol.*, **113**, 173–185.

GAMALLO, C., PALACIOS, J., SUAREZ, A., PIZARRO, A., NAVARRO, P., QUINTANILLA, M. & CANO, A. (1993). Correlation of E-cadherin expression with differentiation grade and histological type in breast carcinomas. *Am. J. Pathol.*, **142**, 987–993.

HASHIMOTO, M., NIWA, O., NITTA, Y., TAKEICHI, M. & YOKORO, K. (1989). Unstable expression of E-cadherin adhesion molecules in metastatic ovarian tumor cells. *Jpn. J. Cancer Res.*, **80**, 459–463.

HIRAI, Y., NOSE, A., KOBAYASHI, S. & TAKEICHI, M. (1989). Expression and role of E- and P-cadherin adhesion molecules in embryonic histogenesis. II. Skin morphogenesis. *Development*, **105**, 271–277.

INOUE, M., OGAWA, H., MIYATA, M., SHIOZAKI, H. & TANIZAWA, O. (1992). Expression of E-cadherin in normal, benign, and malignant tissues of female genital organs. *Am. J. Clin. Pathol.*, **98**, 76–80.

KINSELLA, A.R., GREEN, B., LEPTS, G.C., HILL, C.L., BOWIE, G. & TAYLOR, B.A. (1993). The role of the cell–cell adhesion molecule E-cadherin in large bowel tumour cell invasion and metastasis. *Br. J. Cancer*, **67**, 904–909.

KO, C.B., WALTON, S. & KECZKES, K. (1992). Extensive and fatal basal cell carcinoma: a report of three cases. *Br. J. Dermatol.*, **127**, 164–167.

LEFFELL, D.J., HEADINGTON, J.T., WONG, D.S. & SWANSON, N.A. (1991). Aggressive-growth basal cell carcinoma in young adults. *Arch. Dermatol.*, **127**, 1663–1667.

LEVER, W.F. & SCHAUMBURG-LEVER, G. (1990). *Histopathology of the Skin*, 7th edn. J.B. Lippincott: Philadelphia.

LO, J.S., SNOW, S.N., REIZNER, G.T., MOHS, F.E., LARSON, P.O. & HRUZA, G.J. (1991). Metastatic basal cell carcinoma: report of twelve cases with a review of the literature. *J. Am. Acad. Dermatol.*, **24**, 715–719.

MAGEE, A.I. & BUXTON, R.S. (1991). Transmembrane molecular assemblies regulated by the greater cadherin family. *Curr. Opin. Cell Biol.*, **3**, 854–861.

MAREEL, M.M., BEHRENS, J., BIRCHMEIER, W., DEBRUYNE, G.K., VLEMINCKX, K., HOOGEWUS, A., FIERS, W.C. & VAN ROY, F.M. (1991). Down-regulation of E-cadherin expression in Madin Darby canine kidney (MDCK) cells inside tumors of nude mice. *Int. J. Cancer*, **47**, 922–928.

MATSUURA, K., KAWANISHI, J., FIJII, S., IMAMURA, M., HIRANO, S., TAKEICHI, M. & NIITSU, Y. (1992). Altered expression of E-cadherin in gastric cancer tissues and carcinomatous fluid. *Br. J. Cancer*, **66**, 1122–1130.

MATSUYOSHI, N., HAMAGUCHI, M., TANIGUCHI, S., NAGAFUCHI, A., TSUKITA, S. & TAKEICHI, M. (1992). Cadherin-mediated cell–cell adhesion is perturbed by v-*src* tyrosine phosphorylation in metastatic fibroblasts. *J. Cell Biol.*, **118**, 703–714.

MAYER, B., JOHNSON, J.P., LEITL, F., JAUCH, K.W., HEISS, M.M., SCHILDBERG, F.W., BIRCHMEIER, W. & FUNKE, I. (1993). E-cadherin expression in primary and metastatic gastric cancer. down regulation correlates with cellular differentiation and glandular disintegration. *Cancer Res.*, **53**, 1690–1695.

MILLER, S.J. (1991). Biology of basal cell carcinoma (Part I). *J. Am. Acad. Dermatol.*, **24**, 1–13.

NAVARRO, P., GOMEZ, M., PIZARRO, A., GAMALLO, C., QUINTANILLA, M. & CANO, A. (1991). A role for the E-cadherin cell–cell adhesion molecule during tumor progression of mouse epidermal carcinogenesis. *J. Cell Biol.*, **115**, 517–533.

NICHOLSON, L.J., PEI, X.F. & WATT, F.M. (1991). Expression of E-cadherin, P-cadherin and involucrin by normal and neoplastic keratinocytes in culture. *Carcinogenesis*, **12**, 1345–1349.

OKA, H., SHIOZAKI, H., KOBAYASHI, K., TAHARA, H., TAMURA, S., MIYATA, M., DOKI, Y., IIHARA, K., MATSUYOSHI, N., HIRANO, S., TAKEICHI, M. & MORI, T. (1992). Immunohistochemical evaluation of E-cadherin adhesion molecule expression in human gastric cancer. *Virchows Archiv. A Pathol. Anat.*, **421**, 149–156.

OKA, H., SHIOZAKI, H., KOBAYASHI, K., INOUE, M., TAHARA, H., KOBAYASHI, T., TAKATSUKA, Y., MATSUYOSHI, N., HIRANO, S., TAKEICHI, M. & MORI, T. (1993). Expression of E-cadherin cell adhesion molecules in human breast cancer tissues and its relationship to metastasis. *Cancer Res.*, **53**, 1696–1701.

PIGNATELLI, M., LIU, D., NASIM, M.M., STAMP, G.W.H., HIRANO, S. & TAKEICHI, M. (1992). Morphoregulatory activities of E-cadherin and beta-1 integrins in colorectal tumour cells. *Br. J. Cancer*, **66**, 629–634.

RASBRIDGE, S.A., GILLETT, C.E., SAMPSON, S.A., WALSH, F.S. & MILLIS, R.R. (1993). Epithelial (E-) and placental (P-) cadherin cell adhesion molecule expression in breast carcinoma. *J. Pathol.*, **169**, 245–250.

RUGGERI, B., CAAMANO, J., SLAGA, T.J., CONTI, C.J., NELSON, W.J. & KLEIN-SZANTO, A.J.P. (1992). Alterations in the expression of uvomorulin and $Na^+$, $K^+$-adenosine triphosphatase during mouse skin tumor progression. *Am. J. Pathol.*, **140**, 1179–1185.

SCHIPPER, J.H., FRIXEN, U.H., BEHRENS, J., UNGER, A., JAHNKE, K. & BIRCHMEIER, W. (1991). E-cadherin expression in squamous cell carcinomas of head and neck: inverse correlation with tumor dedifferentiation and lymph node metastasis. *Cancer Res.*, **51**, 6328–6337.

SCHOENENBERGER, C.A. & MATLIN, K.S. (1991). Cell polarity and epithelial oncogenesis. *Trends Cell Biol.*, **1**, 87–92.

SHIMOYAMA, Y. & HIROHASHI, S. (1991*a*). Expression of E- and P-cadherin in gastric carcinomas. *Cancer Res.*, **51**, 2185–2192.

SHIMOYAMA, Y. & HIROHASHI, S. (1991*b*). Cadherin intercellular adhesion molecule in hepatocellular carcinomas: loss of E-cadherin expression in an undifferentiated carcinoma. *Cancer Lett.*, **57**, 131–135.

SHIMOYAMA, Y., HIROHASHI, S., HIRANO, S., NOGUCHI, M., SHIMOSATO, Y., TAKEICHI, M. & ABE, O. (1989). Cadherin cell-adhesion molecules in human epithelial tissues and carcinomas. *Cancer Res.*, **49**, 2128–2133.

SHIMOYAMA, Y., NAGAFUCHI, A., FUJITA, S., GOTOH, M., TAKEICHI, M., TSUKITA, S. & HIROHASHI, S. (1992). Cadherin dysfunction in a human cancer cell line: possible involvement of loss of alpha-catenin expression in reduced cell–cell adhesiveness. *Cancer Res.*, **52**, 5770–5774.

SHIOZAKI, H., TAHARA, H., OKA, H., MIYATA, M., KOBAYASHI, K., TAMURA, S., IIHARA, K., DOKI, Y., HIRANO, S., TAKEICHI, M. & MORI, T. (1991). Expression of immunoreactive E-cadherin adhesion molecules in human cancers. *Am. J. Pathol.*, **139**, 17–23.

SIEGLE, R.J., MACMILLAM, J. & POLLACK, S.V. (1986). Infiltrative basal cell carcinoma: a nonsclerosing subtype. *J. Dermatol. Surg. Oncol.*, **12**, 830–836.

STAMP, G.W.H. & PIGNATELLI, M. (1991). Distribution of beta-1, alpha-1, alpha-2 and alpha-3 integrin chains in basal cell carcinomas. *J. Pathol.*, **163**, 307–313.

TAKEICHI, M. (1991). Cadherin cell adhesion receptors as a morphogenetic regulator. *Science*, **251**, 1451–1455.

TERPE, H.J., TAJROBEHKAR, K., GÜNTHERT, U. & ALTMANNSBERGER, M. (1993). Expression of cell adhesion molecules alpha-2, alpha-5 and alpha-6 integrin, E-cadherin, N-CAM and CD-44 in renal carcinomas. An immunohistochemical study. *Virchows Archiv. A Pathol. Anat.*, **422**, 219–224.

UMBAS, R., SCHALKEN, J.A., AALDERS, T.W., CARTER, B.S., KARTHAUS, H.F.M., SCHAAFSMA, H.E., DEBRUYNE, F.M.J. & ISAACS, W.B. (1992). Expression of the cellular adhesion molecule E-cadherin is reduced or absent in high-grade prostate cancer. *Cancer Res.*, **52**, 5104–5109.

VAN DER WURFF, A.A.M., KATE, J.T., VAN DER LINDEN, E.P.M., DINJENS, W.N.M., ARENDS, J.W. & BOSMAN, F.T. (1992). L-CAM expression in normal, premalignant and malignant colon mucosa. *J. Pathol.*, **168**, 287–291.

VAN ROY, F. & MAREEL, M. (1992). Tumour invasion: effects of cell adhesion and motility. *Trends Cell Biol.*, **2**, 163–169.

VLEMINCKX, K., VAKAET, L., MAREEL, M., FIERS, W. & VAN ROY, F. (1991). Genetic manipulation of E-cadherin expression by epithelial tumor cells reveals an invasion suppressor role. *Cell*, **66**, 107–119.

WHEELOCK, M.J. & JENSEN, P.J. (1992). Regulation of keratinocyte intercellular junction organization and epidermal morphogenesis by E-cadherin. *J. Cell Biol.*, **117**, 415–425.